# Safety and Immunogenicity of Newborn MVA85A Vaccination and Selective, Delayed Bacille Calmette-Guerin for Infants of Human Immunodeficiency Virus-Infected Mothers: A Phase 2 Randomized, Controlled Trial

**DOI:** 10.1093/cid/cix834

**Published:** 2017-10-26

**Authors:** Elisa Nemes, Anneke C Hesseling, Michele Tameris, Katya Mauff, Katrina Downing, Humphrey Mulenga, Penelope Rose, Marieke van der Zalm, Sharon Mbaba, Danelle Van As, Willem A Hanekom, Gerhard Walzl, Thomas J Scriba, Helen McShane, Mark Hatherill, MVA029 Study Team, Charmaine Abrahams, Deborah Abrahams, Hadn Africa, Veronica Baartman, Beauty Bavuma, Nicole Bilek, Natasja Botes, Yolande Brown, Yolundi Cloete, Margareth Damons, Ronel De Vos, Portia Dlakavu, Karen Du Preez, Mzwandile Erasmus, Claudia Francis, Hendrik Geldenhuys, Mandy Geldenhuys, Katriena Goedeman, Sandra Golliath, Angelique Hendricks Mouton, Christiaan Hopley, Ruwijda Jansen, Carolynne Jones, Alana Keyser, Benjamin Kagina, Gloria Khomba, Fazlin Kola–Cassiem, Sandra Kruger, Daphne Leukes, Loyiso Louw, Angelique Luabeya, Theresa Maart, Lebohang Makhethe, Simbarashe Mbabwe, Eunice Mtshamba, Boniswa Mvinjelwa, Lungisa Nkantsu, Julia Noble, Sizwe Nqweniso, Fajwa Opperman, Christel Petersen, Patiswa Plaatjie, Susan Rossouw, Roxanne Solomoms, Marcia Steyn, Liticia Swanepoel, Asma Toefy, Heidi van Deventer, Elma van Rooyen, Daphne van Ster, Bongiwe Vazana, Ashley Veldsman, Noncedo Xoyana

**Affiliations:** 1 South African Tuberculosis Vaccine Initiative, Institute of Infectious Disease & Molecular Medicine and Division of Immunology, Department of Science & Technology/National Research Foundation, University of Cape Town; 2 Desmond Tutu Tuberculosis Centre, Department of Paediatrics and Child Health, Faculty of Medicine and Health Sciences; 3 DST/NRF Centre of Excellence for Biomedical Tuberculosis Research/Medical Research Council Centre for Molecular and Cellular Biology, Division of Molecular Biology and Human Genetics, Faculty of Medicine and Health Sciences, Stellenbosch University, Tygerberg, South Africa; 4 Jenner Institute, Oxford University, United Kingdom

**Keywords:** HIV-exposed infants, MVA85A, BCG, vaccination, tuberculosis

## Abstract

**Background:**

Vaccination of human immunodeficiency virus (HIV)-infected infants with bacille Calmette-Guérin (BCG) is contraindicated. HIV-exposed newborns need a new tuberculosis vaccination strategy that protects against tuberculosis early in life and avoids the potential risk of BCG disease until after HIV infection has been excluded.

**Methods:**

This double-blind, randomized, controlled trial compared newborn MVA85A prime vaccination (1 × 10^8^ PFU) vs Candin^®^ control, followed by selective, deferred BCG vaccination at age 8 weeks for HIV-uninfected infants and 12 months follow-up for safety and immunogenicity.

**Results:**

A total of 248 HIV-exposed infants were enrolled. More frequent mild–moderate reactogenicity events were seen after newborn MVA85A vaccination. However, no significant difference was observed in the rate of severe or serious adverse events, HIV acquisition (n = 1 per arm), or incident tuberculosis disease (n = 5 MVA85A; n = 3 control) compared to the control arm. MVA85A vaccination induced modest but significantly higher Ag85A-specific interferon gamma (IFNγ)+ CD4+ T cells compared to control at weeks 4 and 8 (*P* < .0001). BCG did not further boost this response in MVA85A vaccinees. The BCG-induced Ag85A-specific IFNγ+ CD4+ T-cell response at weeks 16 and 52 was of similar magnitude in the control arm compared to the MVA85A arm at all time points. Proliferative capacity, functional profiles, and memory phenotype of BCG-specific CD4 responses were similar across study arms.

**Conclusions:**

MVA85A prime vaccination of HIV-exposed newborns was safe and induced an early modest antigen-specific immune response that did not interfere with, or enhance, immunogenicity of subsequent BCG vaccination. New protein-subunit and viral-vectored tuberculosis vaccine candidates should be tested in HIV-exposed newborns.

**Clinical Trials Registration:**

NCT01650389.

## Teaser

Newborn MVA85A prime vaccination was safe and induced an early immune response that did not interfere with immunogenicity of subsequent bacille Calmette-Guérin vaccination. New tuberculosis vaccine candidates should be tested using this strategy, which appears safe regardless of infant human immunodeficiency virus exposure.

Bacille Calmette-Guérin (BCG) vaccination of infants remains a key tool to protect young children against tuberculosis [1]. Given young children’s high risk of progression from *Mycobacterium tuberculosis* infection to disease and disseminated forms of tuberculosis, which is associated with severe morbidity and mortality, tuberculosis prevention strategies are of great importance in this population [2, 3]. Infant BCG vaccination offers partial protection against pulmonary, miliary, and meningitic tuberculosis in children [4, 5].

In settings with high tuberculosis burden, all children born to human immunodeficiency virus (HIV)-infected mothers are at increased risk of tuberculosis, including those who remain HIV uninfected [6–8]. A safe and effective tuberculosis vaccine for infants with perinatal HIV exposure is needed urgently, since BCG vaccination of infants known to be HIV infected is contraindicated due to the risk of local, regional, and disseminated BCG disease as well as BCG immune reconstitution inflammatory syndrome following antiretroviral therapy (ART) initiation [9–13]. However, delay in BCG vaccination to allow exclusion of perinatal HIV acquisition would put infants at risk of acquiring tuberculosis in the first weeks of life, in the period before BCG could be administered without safety concerns. These competing risks and benefits have resulted in a pragmatic approach to continued BCG vaccination of HIV-exposed newborns whose HIV infection status is not yet known in settings where rates of childhood tuberculosis and maternal HIV infection are high [13, 14]. For example, approximately one fifth of South African women of reproductive age were HIV infected in 2017 [15]. Despite recent reductions in perinatal HIV transmission [16], the HIV infection rate at age 18 months is considerably higher than at 8 weeks due to high-risk mixed feeding practices [17]. There were an estimated 320 000 South African children living with HIV in 2016 [18]; 50% of deaths among children aged <5 years were associated with HIV infection [19]. Although early HIV polymerase chain reaction (PCR) testing is being introduced, this advance does not solve the BCG safety dilemma because routine BCG is usually given at birth. Also, since HIV-exposed infants in sub-Saharan Africa are often exclusively breast fed, HIV infection may be acquired subsequent to negative PCR testing at age 2 weeks.

Given the high risk of both tuberculosis and BCG-associated adverse events (AEs) in HIV-infected infants, we hypothesized that delaying routine newborn BCG vaccination until HIV infection had been excluded, but preceded by a novel tuberculosis vaccine given at birth, would be safe and more immunogenic than delayed BCG vaccination alone for HIV-exposed infants [20]. We previously showed that delayed BCG vaccination of HIV-unexposed South African infants induces a long-lasting polyfunctional T-cell response, with higher frequencies and better quality of BCG-specific CD4 T cells at age 1 year compared to newborn BCG vaccination [21]. Conflicting studies have shown no significant immunological benefit of delayed BCG [22–24]. However, in utero exposure to maternal HIV and *M. tuberculosis* infection does not appear to alter long-term immune responses of HIV-uninfected infants to BCG vaccination when given at age 6 weeks [25, 26].

New tuberculosis vaccine candidates in clinical development include recombinant and live-attenuated mycobacterial vaccines and viral-vectored or protein-subunit vaccines [27]. A nonreplicating vaccine with a track record of safety in infants and HIV-infected persons would be required to test this novel strategy. Although MVA85A did not confer additional protection when given as a boost vaccine after BCG prime in HIV-uninfected infants [28], MVA85A is an ideal candidate vaccine to test this experimental strategy since MVA85A was safe in this high-risk population [29–33].

Here, we report on the safety and immunogenicity of MVA85A vaccination in newborns of HIV-infected mothers, followed by selective deferred BCG vaccination at 8 weeks for HIV-uninfected infants, in a double-blind, randomized, controlled trial.

## METHODS

The trial was conducted at 2 sites near Cape Town, South Africa. Mothers provided written antenatal and postnatal consent for infant participation. The protocol was approved by the ethics committees of the universities of Cape Town (013/2012), Stellenbosch (M12/03/020), and Oxford (02-12). 

**Figure 1.**
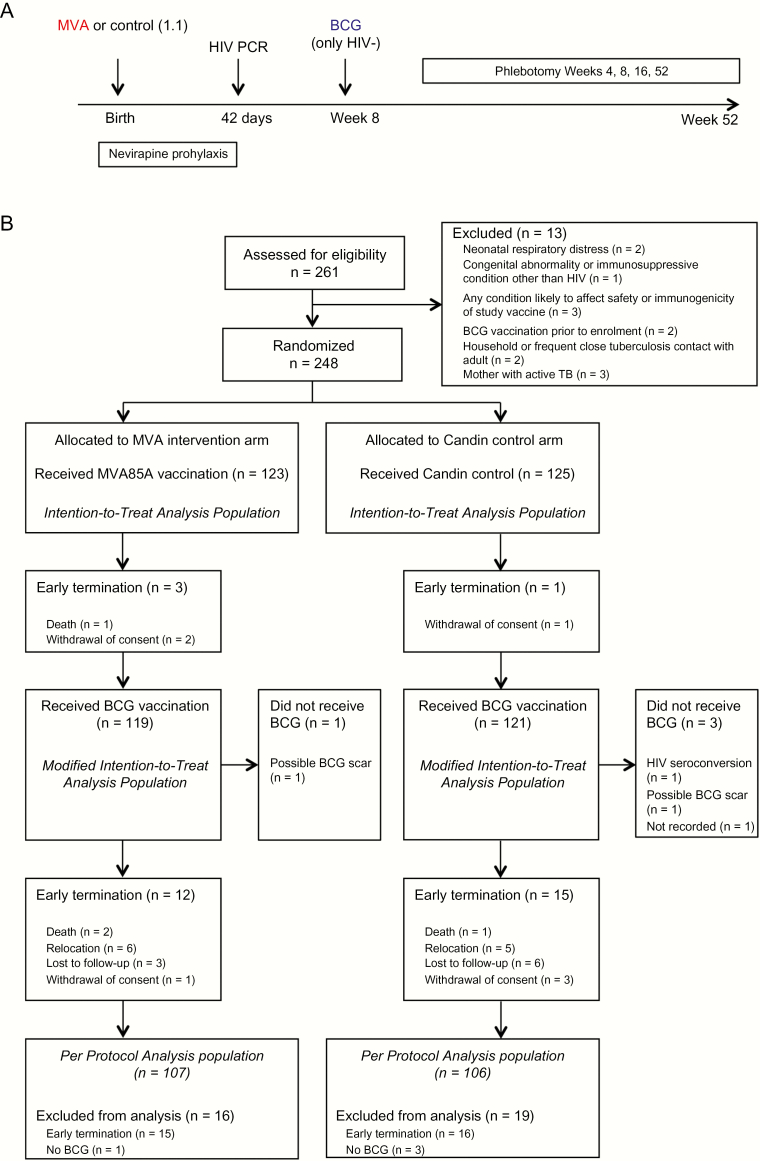
Study design (*A*) and CONSORT diagram (*B*). Abbreviations: BCG, bacille Calmette-Guérin; CONSORT, Consolidated Standards of Reporting Trials; HIV, human immunodeficiency virus; PCR, polymerase chain reaction; TB, tuberculosis.

Eligible infants (see Supplementary Materials) were randomized 1:1 to receive either MVA85A vaccine (1 × 10^8^ PFU) or Candin^®^ control within 96 hours of birth in blinded fashion (Figure 1A). BCG was administered (1–4 × 10^5^ cfu) at age 8 weeks only to infants documented to be HIV-uninfected at age 6 weeks by negative HIV DNA PCR (Roche Diagnostic COBAS AmpliPrep COBAS Taqman HIV-1 Qual test version 2.0)[Figure 1A]. HIV DNA PCR testing prior to age 6 weeks was not routine at the time. All infants were followed for safety endpoints at weeks 1, 4, 6, and 8 after MVA85A/control vaccination and thereafter at weeks 9, 12, and 16 (corresponding to weeks 1, 4, and 8 following delayed BCG vaccination at 8 age weeks) and at week 52. All infants underwent safety monitoring for solicited and unsolicited local, regional, and systemic AEs. For immunogenicity analyses, blood was collected at weeks 4, 8, 16, and 52 (Figure 1A).

QuantiFERON-TB Gold (QFT, Qiagen) was performed on mothers at enrollment of newborns and on infants at age 1 year. Infants who reported a new household tuberculosis contact or developed symptoms or signs of tuberculosis were investigated for tuberculosis as previously described [28]. HIV-uninfected infants with a household tuberculosis contact or positive QFT or tuberculin skin test were referred for isoniazid preventive therapy after exclusion of active tuberculosis. All infants diagnosed with tuberculosis started tuberculosis treatment for disease as per national guidelines.

### Whole Blood Functional Assays

Venous blood was collected in sodium heparin–containing tubes for short- (12 hours) and long-term (7 days) functional assays to measure antigen-specific T-cell responses and proliferative capacity, respectively. Whole blood was left unstimulated (negative control) or stimulated with Ag85A peptide pool, BCG, and phytohemagglutinin (positive control). Assays were conducted as previously described [34, 35] (see Supplementary Materials). Samples were stained with optimized panels of monoclonal antibodies (Supplementary Materials, Table S1) and analyzed using flow cytometry (Supplementary Materials, Figures S1 and S2).

### Statistical Analyses

In the intention-to-treat (ITT) population for safety analysis, the number of AEs was compared between study arms for the periods prior to and following BCG vaccination and for the entire observation period. In each period, counts and percentages of AEs were determined per arm, per category of interest. Tests of proportions per category were used to compare the number of AEs between arms for each type of AE and categories. The Bonferroni correction was applied to account for multiple testing.

Linear mixed-effect models were used to assess the impact of study arm and time and their interaction on the (logged) frequencies of cytokine-producing or cytokine-proliferating T cells. In cases where the distributional assumptions of the model were not met, nonparametric tests were used to assess differences between arms at each time point and changes over time between arms (Wilcoxon rank sum test) and changes over time within arms (Wilcoxon signed rank test).

The ITT population for safety analysis included all infants who received either MVA85A or control. The modified ITT (mITT) population for immunology analysis included all infants who received either MVA85A or control and BCG vaccine and who were not HIV infected, including data from all available sampling time points.

## RESULTS

### Participants

After screening 261 infants, 248 were randomized and included in the ITT safety analysis; 213 infants were analyzed per protocol (Figure 1B). MVA85A and BCG immunogenicity was assessed in 65 infants in the mITT population who received MVA85A (n = 32) or control (n = 33) at birth and BCG at age 8 weeks (n = 65). The median age of infants’ mothers was 28 years; 80% of mothers were receiving long-term ART with median CD4 cell count 424 cells/mm^3^; and 43% of mothers tested QFT positive. Median gestational age of infants was 40 weeks; 48% were male; median birth weight was 3.2 kg; and 61% were breastfed as the initial feeding choice. Baseline demographics by study arm are shown in Table 1.

**Table 1.** Baseline Demographics

Characteristic	MVA85A (n = 123)	Candin^®^ Control (n = 125)
Median birth weight, g (IQR)	3220 (2970–3420)	3170 (2880–3410)
Median gestational age, weeks (IQR)	39 (39–40)	40 (39–40)
Gender female, n (%)	63 (51)	64 (51)
Breastfed, n (%)	70 (57)	75 (60)
Median maternal age, years (IQR)	29 (26–32)	28 (25–33)
Mother receiving antiretroviral therapy, n (%)	98 (80)	101 (81)
Median maternal CD4 count, cells/mm^3^ (IQR)	442 (306–607)	400 (262–554.5)
Maternal QuantiFERON-tuberculosis Gold + n (%)	51 (42)	55 (44)

Abbreviation: IQR, interquartile range.

### Safety

All AEs are shown in Table 2. At least 1 AE was experienced by 243 infants including 239 infants with injection site AEs. The majority of infants experienced mild or moderate AEs. Twenty-five infants experienced at least 1 severe AE, with no difference in rate between the MVA85A (n = 11) and control (n = 14) arms. No life-threatening AEs were observed. Fifty-eight infants had at least 1 serious AE (SAE; n = 26 MVA85A; n = 32 control), including 4 deaths (n = 3 MVA85A; n = 1 control), none of which were classified as related to the investigational product. SAE diagnoses reflected the pattern of respiratory and gastroenteritic illnesses typically observed in the study communities.

**Table 2.** All Adverse Events in the Intention-to-Treat Population Throughout Follow-up

Variable	Total, n (%)	MVA85A, n (%)	Candin^®^ Control, n (%)	*P* Value
Participants with ≥1 adverse event	243 (98)	122 (99.2)	121 (96.8)	
Category				
Injection site	239 (96.4)	121 (98.4)	118 (94.4)	.094
Lymphadenopathy	13 (5.2)	4 (3.3)	9 (7.2)	.163
Systemic	210 (84.7)	106 (86.2)	104 (83.2)	.515
Laboratory	27 (10.9)	14 (11.4)	13 (10.4)	.804
Body system				
Cardiovascular	3 (1.2)	1 (0.8)	2 (1.6)	
Digestive	74 (29.8)	35 (28.5)	39 (31.2)	
Endocrine	15 (6)	6 (4.9)	9 (7.2)	
Hematologic/lymphatic	21 (8.5)	10 (8.1)	11 (8.8)	
Metabolic/nutritional	80 (32.3)	42 (34.1)	38 (30.4)	
Musculoskeletal	1 (0.4)	1 (0.8)	0 (0)	
Neurological	69 (27.8)	34 (27.6)	35 (28)	
Respiratory	87 (35.1)	42 (34.1)	45 (36)	
Skin	241 (97.2)	121 (98.4)	120 (96)	.259
Urogenital	5 (2)	2 (1.6)	3 (2.4)	
Severity				
Mild	243 (98)	122 (99.2)	121 (96.8)	.181
Moderate	116 (46.8)	62 (50.4)	54 (43.2)	.255
Severe	25 (10.1)	11 (8.9)	14 (11.2)	.555
Life-threatening	0 (0)	0 (0)	0 (0)	
Vaccine relationship (MVA85A/control)				
Not related	236 (95.2)	116 (94.3)	120 (96)	
Unlikely	98 (39.5)	50 (40.7)	48 (38.4)	
Possible	66 (26.6)	41 (33.3)	25 (20)	
Probable	9 (3.6)	7 (5.7)	2 (1.6)	
Definite	138 (55.6)	105 (85.4)	33 (26.4)	
Vaccine relationship (bacille Calmette-Guérin)				
Not related	229 (92.3)	122 (99.2)	107 (85.6)	
Unlikely	55 (22.2)	28 (22.8)	27 (21.6)	
Possible	31 (12.5)	14 (11.4)	17 (13.6)	
Probable	4 (1.6)	2 (1.6)	2 (1.6)	
Definite	228 (91.9)	110 (89.4)	118 (94.4)	
Outcome				
Death	4 (1.6)	3 (2.4)	1 (0.8)	
Ongoing	219 (88.3)	106 (86.2)	113 (90.4)	
Recovered with sequelae	24 (9.7)	14 (11.4)	10 (8)	
Recovered without sequelae	238 (96)	121 (99.4)	117 (93.6)	
Unknown	2 (0.8)	1 (0.8)	1 (0.8)	
Seriousness				
Serious	58 (23.4)	26 (21.1)	32 (25.6)	
Not serious	243 (98)	122 (99.2)	121 (96.8)	

Numerator is participants with at least 1 adverse event; denominator is participants in study/arm.

In the 8-week period after newborn MVA85A/control injection and before BCG vaccination, infants in the MVA85A arm were more likely to experience an AE (n = 120) than those in the control arm (n = 84; Table 3). Injection site reactions in this period were more frequent in MVA85A recipients than in controls (n = 119 MVA85A vs n = 32 control; *P* < .0001); there were more AEs among MVA85A recipients that were mild in severity (n = 116 MVA85A vs n = 75 control; *P* < .0001). There was no difference in the rate of AEs, including injection site AEs, after BCG vaccination between study arms (Supplementary Materials, Table S2).

**Table 3.** Adverse Events in the Intention-to-Treat Population Occurring in the Period After MVA85A/Candin^®^ Control Injection and Before bacille Calmette-Guérin Vaccination

Variable	Total, n (%)	MVA85A, n (%)	Candin^®^ Control, |n (%)	*P* Value
Participants with ≥1 adverse event	204 (82.3)	120 (97.6)	84 (67.2)	
Category				
Injection site	151 (60.9)	119 (96.7)	32 (25.6)	<.0001
Lymphadenopathy	11 (4.4)	4 (3.3)	7 (5.6)	.369
Systemic	148 (59.7)	76 (61.8)	72 (57.6)	.501
Laboratory	10 (4)	7 (5.7)	3 (2.4)	.188
Body system				
Cardiovascular	1 (0.4)	0 (0)	1 (0.8)	
Digestive	37 (14.9)	16 (13)	21 (16.8)	
Endocrine	3 (1.2)	2 (1.6)	1 (0.8)	
Hematologic/lymphatic	14 (5.6)	7 (5.7)	7 (5.6)	
Metabolic/nutritional	33 (13.3)	18 (14.6)	15 (12)	
Musculoskeletal	0 (0)	0 (0)	0 (0)	
Neurological	57 (23)	28 (22.8)	29 (23.2)	
Respiratory	28 (11.3)	14 (11.4)	14 (11.2)	
Skin	174 (70.2)	119 (96.7)	55 (44)	<.0001
Urogenital	2 (0.8)	1 (0.8)	1 (0.8)	
Severity				
Mild	191 (77)	116 (94.3)	75 (60)	<.0001
Moderate	67 (27)	43 (35)	24 (19.2)	.005
Severe	10 (4)	4 (3.3)	6 (4.8)	.536
Life-threatening	0 (0)	0 (0)	0 (0)	
Relationship (MVA85A/control)				
Not related	75 (30.2)	37 (30.1)	38 (30.4)	
Unlikely	73 (29.4)	37 (30.1)	36 (28.8)	
Possible	64 (25.8)	40 (32.5)	24 (19.2)	
Probable	9 (3.6)	7 (5.7)	2 (1.6)	
Definite	135 (54.4)	105 (85.4)	30 (24)	
Outcome				
Recovered without sequelae	199 (80.2)	81 (65.9)	118 (94.4)	
Recovered with sequelae	12 (4.8)	6 (4.9)	6 (4.8)	
Ongoing	11 (4.4)	8 (6.5)	3 (2.4)	
Death	1 (0.4)	1 (0.8)	0 (0)	
Unknown	0 (0)	0 (0)	0 (0)	
Seriousness				
Serious	24 (9.7)	10 (8.1)	14 (11.2)	
Not serious	201 (81)	120 (97.6)	81 (64.8)	

Numerator is participants with at least 1 adverse event; denominator is participants in study/arm.

### HIV and *M. tuberculosis* Acquisition, Diagnosis of Tuberculosis Disease

One infant (<1%; control arm) was diagnosed as HIV PCR positive at age 6 weeks and, per protocol, did not receive BCG vaccination at week 8. One (breastfed) infant (<1%; MVA85A arm) was HIV PCR negative at age 6 weeks and received BCG vaccine but subsequently tested HIV PCR positive at age 1 year.

Five infants tested QFT positive at age 1 year (n = 1 MVA85A arm; n = 4 control arm). Eight infants were found to have tuberculosis within the 1-year follow-up period (n = 5 MVA85A; n = 3 control), of whom 1 was *M. tuberculosis* culture positive and 7 were diagnosed on clinical/radiographic grounds and tuberculosis contact history. Two of the tuberculosis cases were QFT positive.

### Ag85A and BCG-Specific T-Cell Responses

To evaluate the immunogenicity of MVA85A and BCG, we measured frequencies of cytokine-producing T cells (expressing combinations of interferon gamma [IFNγ], tumor necrosis factor alpha [TNFɑ], interleukin [IL] 2, IL17, and/or IL22) and their differentiation (based on coexpression of CD45RA and CCR7) after 12-hour stimulation of whole blood with Ag85A or BCG, respectively.

**Figure 2.**
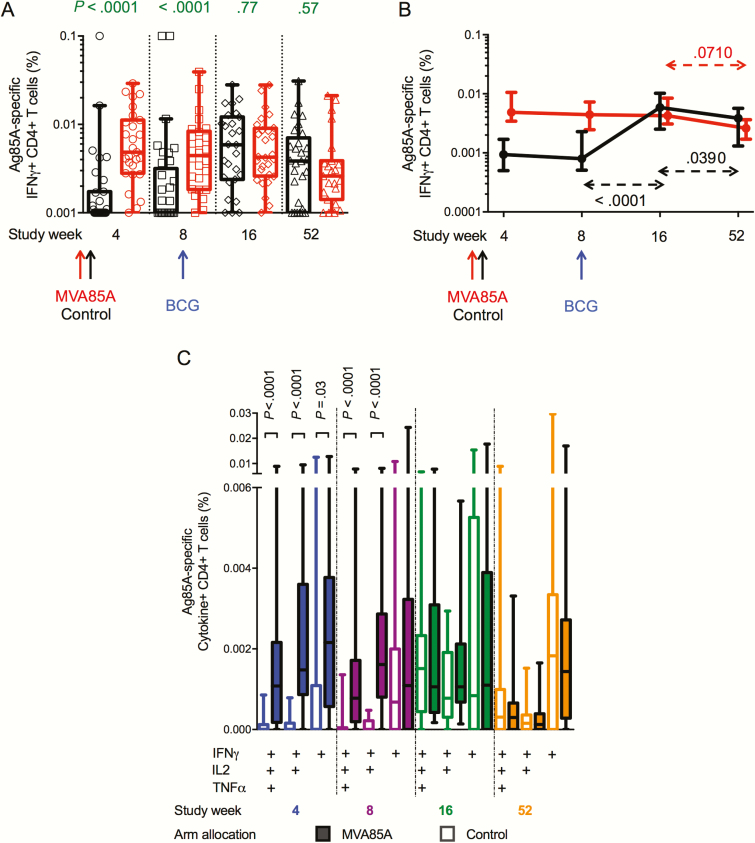
Ag85A-specific CD4 cytokine responses. Fresh whole blood was stimulated with Ag85A peptides for 12 hours prior to intracellular cytokine staining and flow cytometry analysis. *A*, Cross-sectional comparison of frequencies of Ag85A-specific CD4+ T cells expressing interferon gamma (IFNγ) in participants who were vaccinated with MVA85A (red) or control (black) at birth. Bacille Calmette-Guérin was administered to all participants at age 8 weeks. *B*, Longitudinal changes of Ag85A-specific CD4+ T cells expressing IFNγ are indicated by arrows (red for MVA85A arm and black for control arm). Medians and 95% confidence intervals (for the medians) are shown. *C*, Frequencies of Ag85A-specific CD4+ T cells expressing different combinations of IFNγ, tumor necrosis factor alpha, and interleukin 2 were compared between MVA85A arm (solid boxes) and control arm (clear boxes) at weeks 4 (blue), 8 (purple), 16 (green), and 52 (orange). Box and whiskers denote median, interquartile range, and minimum/maximum. Unadjusted *P* values were calculated by mixed effects models in *A* and *B* and by Mann-Whitney test in *C*. Abbreviations: BCG, bacille Calmette-Guérin; IFNγ, interferon gamma; IL, interleukin; TNFα, tumor necrosis factor alpha.

MVA85A induced higher frequencies of Ag85A-specific IFNγ+ CD4+ T cells 4 and 8 weeks post-vaccination compared to control (Figure 2A). BCG-induced Ag85A-specific CD4+ T cells in placebo recipients were of similar magnitude to those induced by MVA85A. BCG vaccination did not boost Ag85A-specific CD4+ T cells induced by MVA85A (Figure 2B).

MVA85A induced mainly IFNγ-expressing CD4+ T cells, many of which coexpressed IL2 and TNFɑ (Figure 2C). There was no detectable IL17 or IL22 production by Ag85A-specific CD4+ T cells before BCG vaccination (data not shown).

**Figure 3.**
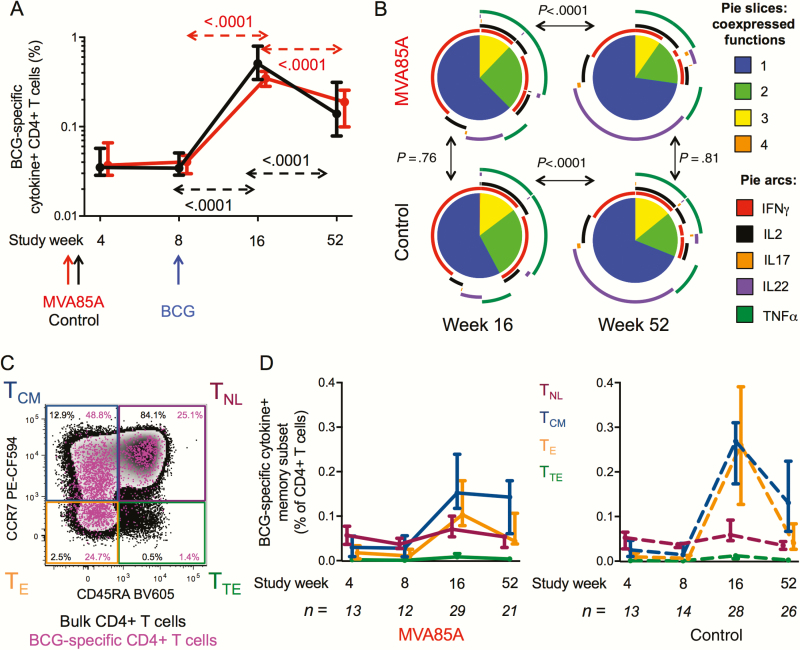
Bacille Calmette-Guérin (BCG)-specific CD4 cytokine responses. Fresh whole blood was stimulated with BCG for 12 hours prior to intracellular cytokine staining and flow cytometry analysis. *A*, Longitudinal changes of BCG-specific CD4+ T cells expressing any combination of interferon gamma (IFNγ), tumor necrosis factor alpha (TNFα), interleukin (IL) 2, IL17, and/or IL22 are indicated by arrows (red for MVA85A arm and black for control arm). Medians and 95% confidence intervals (CIs; for the medians) are shown. Unadjusted *P* values were calculated by mixed effects models. *B*, Cytokine coexpression patterns of BCG-specific CD4 responses at week 16 (left) and week 52 (right) in the MVA85A (top) and control (bottom) arms by permutation test. Pies represent total BCG-specific CD4+ T cells expressing any cytokine; slices show the relative proportion of cells coexpressing 1 (blue), 2 (green), 3 (yellow), or 4 (orange) cytokines, identified by the external arcs: IFNγ (red), IL2 (black), IL17 (orange), IL22 (purple), and TNFα (dark green). *C*, Differentiation profiles were defined based on expression patterns of CD45RA and CCR7 as follows: naive-like (T_NL_, CD45RA+ CCR7+), central memory (T_CM_, CD45RA- CCR7+), effector (T_E_, CD45RA- CCR7-), and terminal effector (T_TE_, CD45RA+ CCR7-). Representative flow cytometry plot of BCG-specific cytokine+ CD4+ T cells (pink) overlaid on total CD4+ T cells (black). *D*, Longitudinal changes of BCG-specific cytokine+ CD4+ T cells expressing T_NL_ (maroon), T_CM_ (blue), T_E_ (orange), or T_TE_ (green) phenotype in MVA85A (left) or control (right) recipients. Medians and 95% CIs (for the medians) are shown. The number of participants meeting cutoff criteria for this analysis (see methods) is shown for each visit. Frequencies of all subsets significantly increased (*P* < .025) upon BCG vaccination (week 8 vs week 16) and decreased (*P* < .025) between week 16 and 52, with the exception of T_CM_ in the MVA85A arm. Unadjusted *P* values were calculated by Wilcoxon matched-pairs test; week 4 and 8 were not compared due to low numbers of paired samples (less than 10). No significant differences were observed when comparing frequencies of each subset at each visit between study arms. Abbreviations: BCG, bacille Calmette-Guérin; IFNγ, interferon gamma; IL, interleukin; TNFα, tumor necrosis factor alpha.

BCG vaccination induced markedly increased and durable CD4+ T-cell responses in the MVA85A prime and control groups (Figure 3A). The cytokine coexpression profiles of BCG-specific CD4+ T cells were different during the effector (week 16) and memory (week 52) phases of the response and were not affected by MVA85A prime (Figure 3B). At week 52 most BCG-specific CD4+ T cells were monofunctional (Figure 3B, blue slice), and the predominant subset of these cells expressed IL22 alone (Figure 3B, purple arc).

We also measured memory phenotype of cytokine-expressing, BCG-specific CD4+ T cells (Figure 3C). Frequencies of naive-like (T_NL_), central memory (T_CM_), effector (T_E_), and terminal effector (T_TE_) BCG-specific CD4+ T cells were not different between study arms at any visit, although BCG administration induced all subsets significantly (week 8 vs 16; *P* < .025 for all subsets in both study arms). Central memory T cells were maintained at similar levels between week 16 and week 52 only in the MVA85A arm, while all other subsets decreased (*P* < .025).

Ag85A- and BCG-specific CD8+ T cells were detected at low levels, predominantly expressed IFNγ or TNFα, and were not different between the study groups (Supplementary Materials, Figure S3A, S3B and data not shown).

### T-Cell Proliferative Responses to Ag85A and BCG

T-cell proliferation is a sensitive measurement of vaccine immunogenicity. BCG-specific proliferative responses are typically not persistently affected by HIV exposure [26] or delayed BCG administration [23]. Long-term recall and effector potential of MVA85A and BCG-induced T-cell responses were assessed by measuring proliferative capacity (expression of cell cycle–associated marker Ki67) of antigen-specific T cells and their cytotoxic potential (upregulation of cytotoxic mediators granzyme A, granzyme B, granzyme K, granulysin, and perforin) upon 7-day stimulation of whole blood with Ag85A or BCG, respectively.

**Figure 4.**
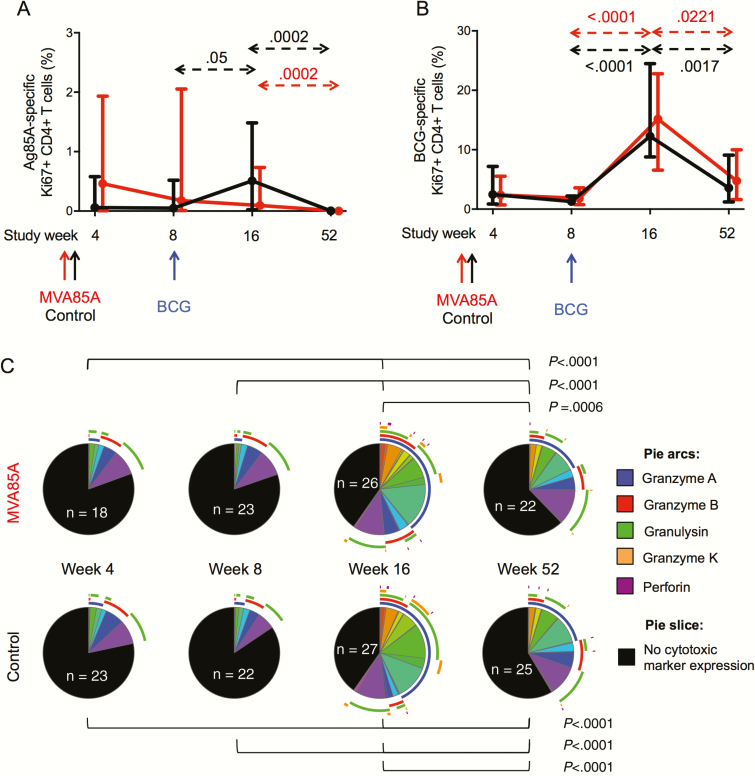
CD4+ T cell proliferation in response to Ag85A and bacille Calmette-Guérin (BCG). Fresh whole blood was stimulated with Ag85A peptides or BCG for 7 days prior to intracellular staining of Ki67 and cytotoxic markers and flow cytometric analysis. Frequencies of Ag85A-specific (*A*) and BCG-specific (*B*) CD4+ T cells expressing the proliferation marker Ki67 were analyzed longitudinally in MVA85A (red lines and arrows) and control (black lines and arrows) arms. Medians and 95% confidence intervals (for the medians) are shown. Unadjusted *P* values were calculated by mixed effects models. *C*, Cytotoxic mediator coexpression patterns of BCG-specific CD4 responses were compared across study weeks in MVA85A arm (top) and control arm (bottom) by permutation test. No significant differences between study arms were observed at any visit. Pies represent total BCG-specific CD4+ T cells expressing Ki67, and slices represent the relative proportion of cells coexpressing cytotoxic markers identified by the external arcs: granzyme A (blue), granzyme B (red), granulysin (green), granzyme K (orange), and perforin (purple). Black slices denote the proportion of proliferating cells that do not express any cytotoxic marker. The number of participants meeting cutoff criteria for this analysis (see methods) is shown within each pie. Abbreviations: BCG, bacille Calmette-Guérin.

MVA85A and BCG induced similar low CD4 proliferative responses to Ag85, which was lost by age 1 year (Figure 4A). BCG administration did not further boost proliferative responses primed by MVA85A.

BCG vaccination induced a strong CD4 proliferative response to BCG, which did not differ by study arm and was not sustained above prevaccination levels by age 1 year (Figure 4B).

During the effector phase of BCG-induced responses (week 16), the majority of proliferating CD4+ T cells upregulated expression of cytotoxic mediators (Figure 4C), mostly granzyme A (blue arc), granzyme B (red arc), and granulysin (green arc). Proportions of proliferating CD4+ T cells expressing cytotoxic mediators had decreased by week 52 but still comprised approximately one third of BCG-specific CD4+ T cells (Figure 4C). No differences in the cytotoxic potential were observed between study arms at any time point (Figure 4C).

## DISCUSSION

Newborn administration of a viral-vectored prime vaccine (MVA85A), followed by BCG vaccine boost at age 8 weeks, had an acceptable safety and reactogenicity profile; induced modest, antigen-specific responses before BCG administration; and did not interfere with or enhance subsequent BCG immunogenicity. These findings demonstrate proof of principle that a novel tuberculosis vaccination strategy based on a newborn priming vaccine other than BCG, including candidates that are potentially more immunogenic than MVA85A, can be administered safely to HIV-exposed newborns. Such a strategy would avoid the risks associated with administration of live BCG vaccine to infants with undiagnosed perinatal HIV infection. We infer from these findings that a new efficacious subunit or viral-vectored tuberculosis vaccine might also be given safely to newborns to provide protection against tuberculosis disease in the early weeks of life.

It is notable that the rate of HIV acquisition (<1%) was low compared to historical perinatal HIV transmission rates in South Africa (2.7% in 2012 [17]). This is due in part to systemic improvements in the perinatal HIV prevention (prevention of mother-to-child transmission [PMTCT]) and because maternal ART or perinatal prophylaxis was a requirement for infant enrollment. Therefore, this alternative tuberculosis vaccination strategy would be expected to have even greater impact on BCG vaccine safety in countries where PMTCT programs are weaker and perinatal HIV transmission rates are higher.

It is also striking that the rate of QFT conversion in HIV-exposed infants at age 1 year (2.5%) was lower than that reported for HIV-unexposed infants in these communities (6%–7%) [28, 36]. While the overall incidence of tuberculosis (3.3%) was similar to what has been described in previous reports [28], clinical diagnoses are likely to have resulted in overestimation of the true disease rate. It is likely that the exclusion criterion for household tuberculosis contact reduced the risk for tuberculosis transmission and disease in study infants, as evidenced by the maternal QFT positive rate (43%), which is considerably lower than that observed in HIV-uninfected young adults in the same community [36].

We and others have shown that deferring BCG administration from birth to age 6–18 weeks does not impair long-term BCG immunogenicity [21–24]. Here, we evaluated the effects of administering a newborn prime tuberculosis vaccine on the immunogenicity of deferred BCG vaccination. MVA85A was weakly immunogenic, inducing mainly IFNγ+ Ag85A-specific cells before BCG administration. In BCG-vaccinated infants, higher abundance of cells releasing IFNγ upon BCG stimulation was associated with lower risk of progression to tuberculosis disease [37]. Whether the low IFNγ+ T-cell responses induced by MVA85A could be sufficient to protect against tuberculosis before BCG administration is unknown. Our findings differ from those in previous observations in HIV-exposed and HIV-unexposed infants [38, 39] in which MVA.HIVA was poorly immunogenic, due possibly to differences in study design, lower dose of MVA.HIVA, age at administration, and the assay used to measure immunogenicity. Nevertheless, these trials showed no interference by MVA administration with immunogenicity of routine childhood vaccines, further supporting the clinical development of this strategy.

Remarkably, BCG did not further boost MVA85A-primed Ag85A-specific T-cell responses. These findings differ from observations made using the converse vaccination strategy, in which MVA85A significantly enhanced Ag85A-specific CD4 T-cell responses primed by BCG [28]. These observations suggest that either MVA85A is more immunogenic when used as a boost vaccine after BCG priming or, alternatively, that BCG cannot further boost Ag85A-specific T-cell responses that have been maximally primed by MVA85A. Regardless, BCG vaccination induced similar magnitudes of Ag85A-specific T-cell responses in the control and MVA85A groups. We deduce that for a given antigen, MVA is as good a vector as BCG, but that the immune response to Ag85A in this newborn population is weak. Importantly, MVA85A prime did not interfere with BCG immunogenicity with respect to the magnitude, functional quality, memory phenotype, and proliferative capacity of antigen-specific CD4+ T cells.

T-cell proliferation was measured to assess relevant immune functions other than cytokine production, such as long-term recall responses and cytotoxic potential, with a more sensitive assay. Unlike cytokine production, T-cell proliferative responses to Ag85A and BCG were not sustained at age 1 year. Similarly, we previously reported that IFNγ release measured upon a 7-day whole blood stimulation with BCG was mostly undetectable by age 1 year, irrespective of age at BCG administration (birth vs 14 weeks) and HIV exposure [40]. While the underlying reasons for the loss of proliferative potential remain to be determined, it is clear that measuring T-cell functions other than IFNγ production is important to assess immunogenicity of novel vaccination strategies.

Interpretation of our findings is limited by the lack of HIV-unexposed and BCG-naive control groups, both of which would not be ethically permissible in a highly tuberculosis-endemic setting. Further, the study sample size was selected to assess safety and immunogenicity and was not powered to test efficacy against *M. tuberculosis* infection or tuberculosis disease. Finally, although MVA85A has an excellent safety track record that is ideal for an experimental medicine study, further studies are needed to test this principle for potentially more efficacious tuberculosis vaccine candidates.

In conclusion, the acceptable safety and reactogenicity profile, modest immunogenicity, and lack of interference with immunogenicity of BCG support further testing of alternative newborn prime vaccines, including other vector-based and protein-adjuvant candidates with additional antigens to enhance immunogenicity. This novel strategy should be pursued in order to provide protective immunity against *M. tuberculosis* in the first months of life, while being safe for all HIV-exposed infants.

## Supplementary Data

Supplementary materials are available at *Clinical Infectious Diseases* online. Consisting of data provided by the authors to benefit the reader, the posted materials are not copyedited and are the sole responsibility of the authors, so questions or comments should be addressed to the corresponding author.

## Supplementary Material

Supplementary Figure 1

Supplementary Figure 2

Supplementary Figure 3

Supplementary Table 1

Supplementary Table 2

Supplementary Information

## Notes

***Acknowledgments***. We thank study participants, their families, and the communities of Cape Winelands East district and Khayelitsha; personnel at the South African Tuberculosis Vaccine Initiative and the Desmond Tutu TB Center; and the study Safety Monitor (Zaynab Waggie) and Data Safety Monitoring Committee (Prakash Jeena, Brian Eley, Lewellys Barker, and Margaret Snowden).

***Financial support***. This work was supported by the UK Medical Research Council, Department for International Development, and Wellcome Trust Joint Global Health Trials programme (grant G1100570/1) and by AERAS. H. Mc. S. is a Wellcome senior clinical research fellow.

***Potential conflicts of interest***. The authors declare no conflict of interest. All authors have submitted the ICMJE Form for Disclosure of Potential Conflicts of Interest. Conflicts that the editors consider relevant to the content of the manuscript have been disclosed.

## Appendix

***MVA029 Study Team***. Charmaine Abrahams, Deborah Abrahams, Hadn Africa, Veronica Baartman, Beauty Bavuma, Nicole Bilek, Natasja Botes, Yolande Brown, Yolundi Cloete, Margareth Damons, Ronel De Vos, Portia Dlakavu, Karen Du Preez, Mzwandile Erasmus, Claudia Francis, Hendrik Geldenhuys, Mandy Geldenhuys, Katriena Goedeman, Sandra Golliath, Angelique Hendricks Mouton, Christiaan Hopley, Ruwijda Jansen, Carolynne Jones, Alana Keyser, Benjamin Kagina, Gloria Khomba, Fazlin Kola–Cassiem, Sandra Kruger, Daphne Leukes, Loyiso Louw, Angelique Luabeya, Theresa Maart, Lebohang Makhethe, Simbarashe Mbabwe, Eunice Mtshamba, Boniswa Mvinjelwa, Lungisa Nkantsu, Julia Noble, Sizwe Nqweniso, Fajwa Opperman, Christel Petersen, Patiswa Plaatjie, Susan Rossouw, Roxanne Solomoms, Marcia Steyn, Liticia Swanepoel, Asma Toefy, Heidi van Deventer, Elma van Rooyen, Daphne van Ster, Bongiwe Vazana, Ashley Veldsman, Noncedo Xoyana.
