# Supplementary figures and images for: Safety and Immunogenicity of Newborn MVA85A Vaccination and Selective, Delayed Bacille Calmette-Guerin for Infants of Human Immunodeficiency Virus-Infected Mothers: A Phase 2 Randomized, Controlled Trial
Source: Clin Infect Dis. 2017 Oct 26;66(4):554–63. doi: 10.1093/cid/cix834 (PMC5849090; doi:10.1093/cid/cix834)

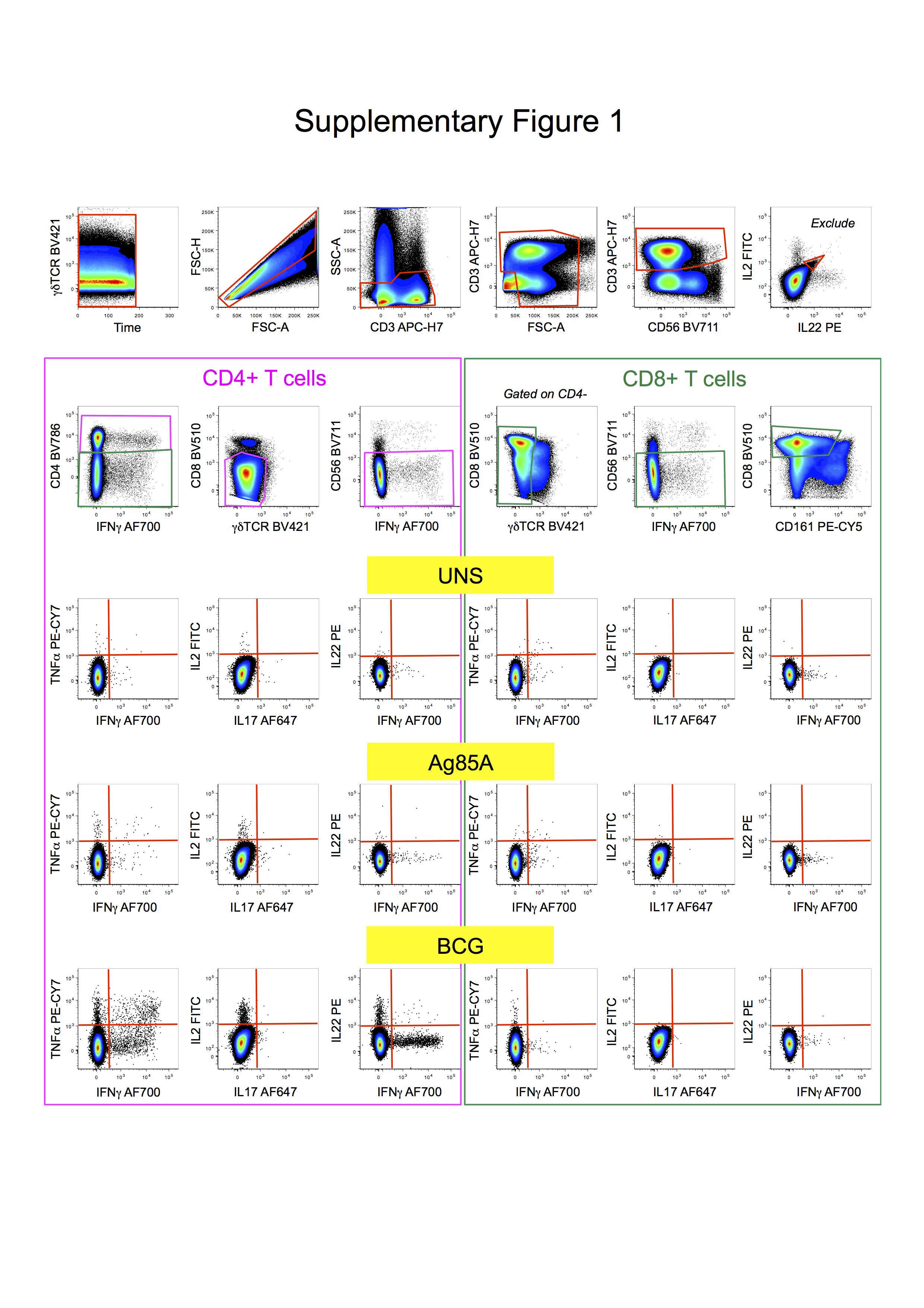

Supplement: Supplementary Figure 1 [file cix834_suppl_suppl_fig.1.png]

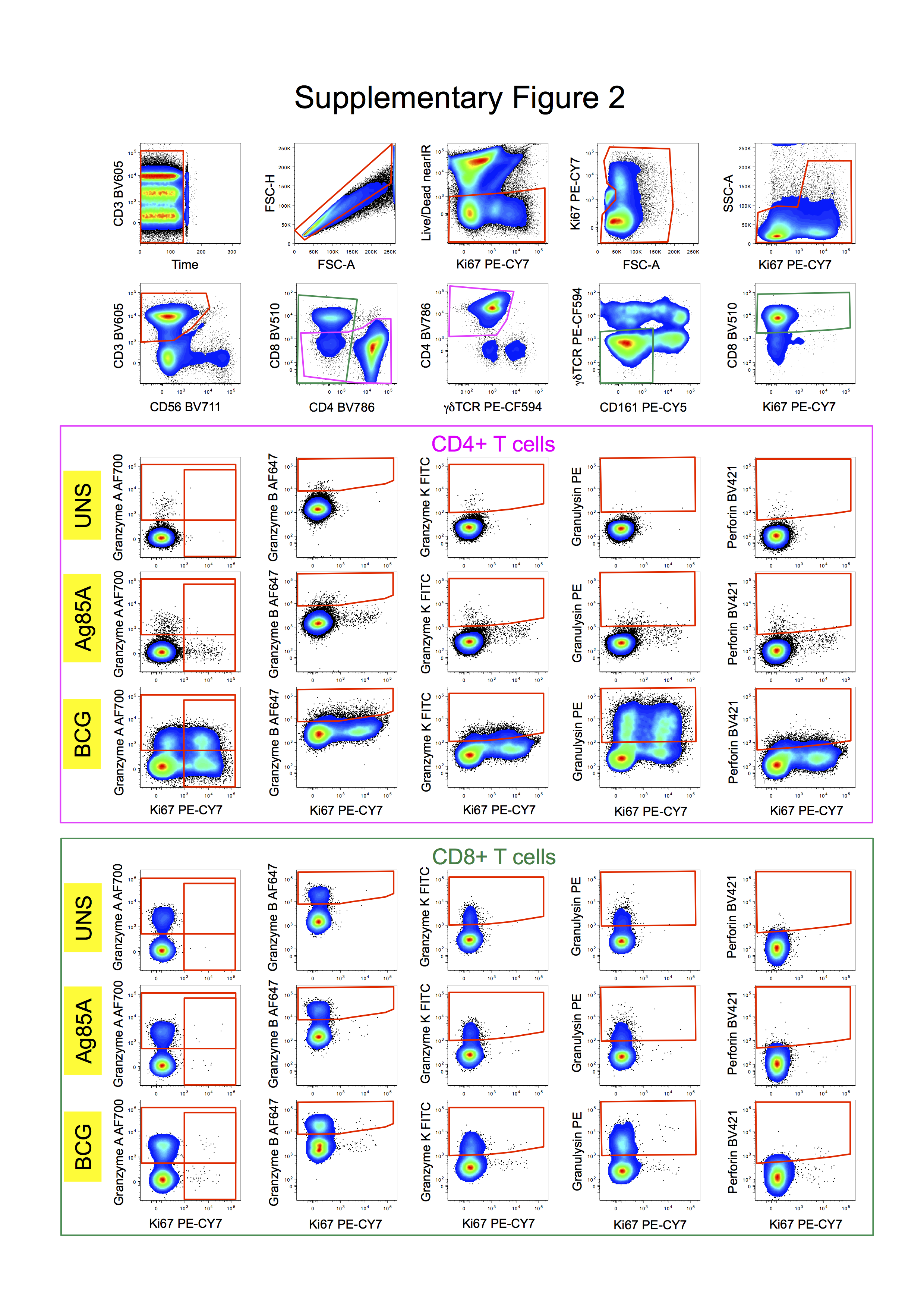

Supplement: Supplementary Figure 2 [file cix834_suppl_suppl_fig.2.png]

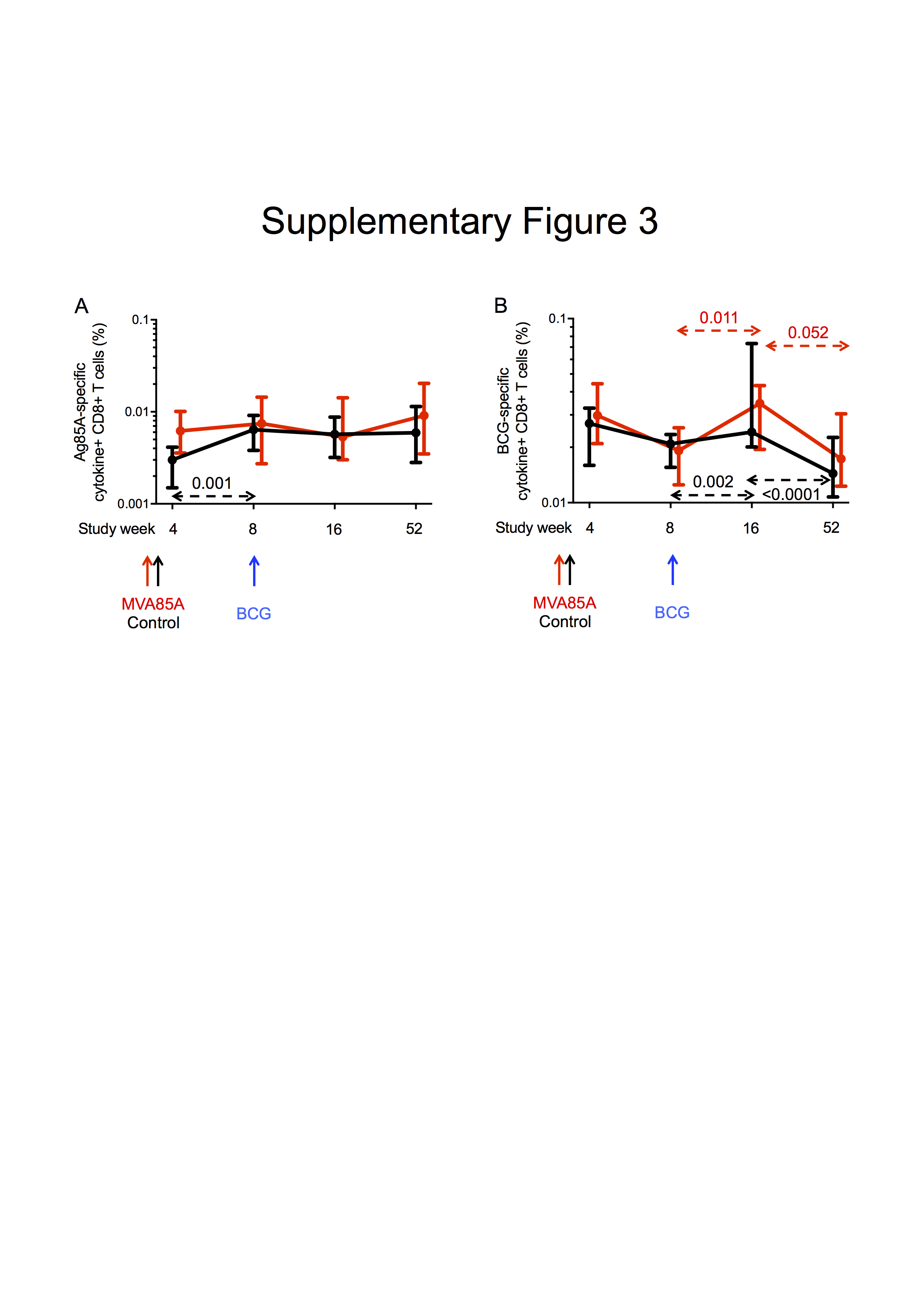

Supplement: Supplementary Figure 3 [file cix834_suppl_suppl_fig.3.png]
