# Supplementary material for: Safety and Immunogenicity of Newborn MVA85A Vaccination and Selective, Delayed Bacille Calmette-Guerin for Infants of Human Immunodeficiency Virus-Infected Mothers: A Phase 2 Randomized, Controlled Trial
Source: Clin Infect Dis. 2017 Oct 26;66(4):554–63. doi: 10.1093/cid/cix834 (PMC5849090; doi:10.1093/cid/cix834)
Supplement: Supplementary Table 2 [file cix834_suppl_supplementary_table2.docx]

**Supplementary Table 2. All AEs in the Intention to Treat (ITT) Population occurring in the period after BCG vaccination**

**(numerator participants with at least one AE; denominator participants in study/arm)**

|  | **Total**  **n (%)** | **MVA85A**  **n (%)** | **Candin® Control**  **n (%)** | **p-value** |
| --- | --- | --- | --- | --- |
| Participants with ≥1 AE | 235 (94.8) | 116 (94.3) | 119 (95.2) |  |
| **CATEGORY** |  |  |  |  |
| Injection site | 227 (91.5) | 110 (89.4) | 117 (93.6) | 0.238 |
| Lymphadenopathy | 2 (0.8) | 0 (0) | 2 (1.6) | 0.159 |
| Systemic | 161 (64.9) | 77 (62.6) | 84 (67.2) | 0.448 |
| Laboratory | 19 (7.7) | 9 (7.3) | 10 (8) | 0.840 |
| **BODY SYSTEM** |  |  |  |  |
| Cardiovascular (C) | 2 (0.8) | 1 (0.8) | 1 (0.8) |  |
| Digestive (D) | 51 (20.6) | 24 (19.5) | 27 (21.6) |  |
| Endocrine (E) | 12 (4.8) | 4 (3.3) | 8 (6.4) |  |
| Haematologic/lymphatic (H) | 10 (4) | 5 (4.1) | 5 (4) |  |
| Metabolic/nutritional (M) | 61 (24.6) | 32 (26) | 29 (23.2) |  |
| Musculoskeletal (MS) | 1 (0.4) | 1 (0.8) | 0 (0) |  |
| Neurological (N) | 30 (12.1) | 11 (8.9) | 19 (15.2) |  |
| Respiratory (R) | 70 (28.2) | 32 (26) | 38 (30.4) |  |
| Skin (S) | 230 (92.7) | 112 (91.1) | 118 (94.4) | 0.310 |
| Urogenital (U) | 3 (1.3) | 1 (0.9) | 2 (1.7) |  |
| **SEVERITY** |  |  |  |  |
| Mild | 235 (94.8) | 116 (94.3) | 119 (95.2) | 0.753 |
| Moderate | 68 (27.4) | 30 (24.4) | 38 (30.4) | 0.289 |
| Severe | 15 (6) | 7 (5.7) | 8 (6.4) | 0.815 |
| Life-threatening | 0 (0) | 0 (0) | 0 (0) |  |
| **VACCINE RELATIONSHIP**  **(BCG Vaccine)** |  |  |  |  |
| Not related | 137 (55.2) | 67 (54.5) | 70 (56) |  |
| Unlikely | 52 (21) | 25 (20.3) | 27 (21.6) |  |
| Possible | 30 (12.1) | 14 (11.4) | 16 (12.8) |  |
| Probable | 4 (1.6) | 2 (1.6) | 2 (1.6) |  |
| Definite | 227 (91.5) | 110 (89.4) | 117 (93.6) |  |
| **OUTCOME** |  |  |  |  |
| Recovered without Sequelae | 220 (88.7) | 110 (89.4) | 110 (88) |  |
| Recovered with Sequelae | 18 (7.3) | 11 (8.9) | 7 (5.6) |  |
| Ongoing | 214 (86.3) | 103 (83.7) | 111 (88.8) |  |
| Death | 3 (1.2) | 2 (1.6) | 1 (0.8) |  |
| Unknown | 2 (0.8) | 1 (0.8) | 1 (0.8) |  |
| **BY SERIOUSNESS** |  |  |  |  |
| Serious | 40 (16.1) | 17 (13.8) | 23 (18.4) |  |
| Not Serious | 235 (94.8) | 116 (94.3) | 119 (95.2) |  |
