# Supplementary material for: Safety and Immunogenicity of Newborn MVA85A Vaccination and Selective, Delayed Bacille Calmette-Guerin for Infants of Human Immunodeficiency Virus-Infected Mothers: A Phase 2 Randomized, Controlled Trial
Source: Clin Infect Dis. 2017 Oct 26;66(4):554–63. doi: 10.1093/cid/cix834 (PMC5849090; doi:10.1093/cid/cix834)
Supplement: Supplementary Information [file cix834_suppl_supplementary_information.docx]

**Supplementary Information**

**Methods**

**Study procedures**

Study staff attended the birth clinic before routine BCG was administered. Infants of mothers known to be HIV infected were enrolled within 96 hours of birth, provided that the mother provided written informed consent; was resident in the study area and able to attend study visits; aged 18 years or older; and receiving either antiretroviral treatment (ART) or prevention of mother to child transmission (PMTCT) HIV prophylaxis. The South African national 2010 PMTCT guideline recommended antenatal Zidovudine; intra-partum single-dose Nevirapine; and 3-hourly AZT; postpartum single dose Tenofovir plus Emtracitabine; with infant Nevirapine prophylaxis and support for feeding choices. Infants were excluded if they had neonatal Apgar score <7 at 5 minutes; birth weight <2,000g or >4,500g; estimated gestational age <32 weeks; neonatal respiratory distress; history or evidence of infant congenital abnormality or immunosuppressive condition other than HIV infection; any other condition likely to affect safety or immunogenicity of study vaccine; received BCG vaccination prior to enrolment; or resided in a household with an adult diagnosed with active TB.

Assignment to study arm was double-blinded and based on a random number sequence prepared by an independent statistician. The study pharmacist, the only unblinded member of the study team, controlled the numbered sealed envelopes containing randomization arm and sequential 3-digit enrolment number.

MVA85A vaccine is a modified vaccinia virus Ankara (MVA), a highly attenuated vaccinia strain that does not replicate in mammalian cells, expressing the *M. tuberculosis* antigen 85A, a highly conserved immuno-dominant mycobacterial antigen. MVA85A has a long track record of safety in infants, children, and adults, including HIV infected persons. The dose of MVA85A vaccine used in this trial was 1x10^8^ pfu. The control immunisation Candin® (Allermed Laboratories Inc., 7203 Conway Court, SanDiego, CA 92111 U.S.A.) was purified skin test antigen for *Candida albicans* of standardized potency, indicated for evaluation of delayed cellular hypersensitivity, administered at the same volume as MVA85A. The study pharmacist prepared blinded study vaccine syringes using aseptic technique for intradermal injection within 96 hours of birth, according to randomization group.

BCG vaccine was administered on the right upper arm in the deltoid region using the Mantoux technique, as per standard of care. The BCG vaccine used in this study (Serum Institut, Copenhagen, Denmark) was the strain used by the South African national vaccination programme, derived from the Danish BCG strain 1331. After reconstitution, 1 dose (0.05 mL) for infants under 12 months of age contains *Mycobacterium bovis* BCG (Bacillus Calmette-Guérin), Danish strain 1331, live attenuated, 1-4 x 10^5^ cfu.

Mothers and infants attended routine PMTCT services for treatment, including nevirapine for 6 weeks after birth or for the duration of breastfeeding if infant HIV uninfected and mother not on ART; with cotrimoxazole beginning at 6 weeks of age and continuing for the duration of breastfeeding. Infants diagnosed HIV infected by PCR at 6 weeks of age continued cotrimoxazole and were referred for ART as per national guidelines. Infants diagnosed HIV-uninfected at 6 weeks of age had HIV PCR repeated at cessation of breastfeeding and as clinically indicated.

Intensified safety and reactogenicity data for MVA85A/control vaccination at birth was collected in a pilot safety cohort (n=60).

**Whole blood functional assays**

For the 12-hour whole blood assay (WBA, [1,2]), whole blood was stimulated within 75 minutes from collection with medium alone (unstimulated), BCG vaccine (Statens Serum Institut, 1,2x10^6^ CFU/mL), Ag85A peptide pool (15 mers overlapping by 10 aminoacids, GenScript, 2 μg/mL/peptide) and PHA (Phytohemagglutinin as positive control, Bioweb, 5 μg/mL) in presence of co-stimulatory antibodies (anti-CD28 and anti-CD49d, BD Biosciences, used both at 0.25 μg/mL). Brefeldin A (Sigma Aldrich, 10 μg/mL) was added after 7 hours of incubation, and samples were further incubated at 37°C for 5 hours. Cells were harvested by adding EDTA (2 mM, Sigma-Aldrich), red blood cells were lysed and white blood cells were fixed using FACSlyse solution (BD) prior to cryopreservation in a solution of RPMI (Lonza), 40% Foetal Calf Serum (FCS, Hyclone), 10% dimethyl sulfoxide (DMSO, Sigma Aldrich).

For the 7-day WBA (adapted from [3]), whole blood was diluted 1:10 in RPMI (Lonza) and incubated within 90 minutes from collection with medium alone (unstimulated), BCG vaccine (Statens Serum Institut, 1x10^5^ CFU/mL), Ag85A peptide pool (15 mers overlapping by 10 aminoacids, GenScript, 0.2 μg/mL/peptide) and PHA (Phytohemagglutinin as positive control, Bioweb, 0.3 μg/mL). Plates were incubated at 37°C for 7 days. Cells were harvested by adding EDTA (2 mM, Sigma-Aldrich), red blood cells were lysed using an ammonium chloride-based solution (1.5M NH_4_Cl, 100mM KHCO_3_, 10mM Na_4_EDTA, Sigma-Aldrich), and white blood cells were stained with near infra-red fixable dead cell dye (Molecular Probes) for 20 minutes at room temperature. Samples were further treated with BD FACSlyse solution and cryopreserved in a solution of RPMI, 40% FCS, 10% DMSO.

**Flow cytometry**

Cryopreserved samples were thawed, permeabilized for 10 minutes at room temperature (BD Perm Wash) and stained for 45-60 minutes at 4°C (CCR7 was stained for 20 minutes at 37°C prior to addition of other antibodies) with optimised panels of pre-titrated monoclonal antibodies (Supplementary table 1) diluted in Brilliant Stain buffer (BD Biosciences). Single-stained antibody capture beads (BD Biosciences) were used to calculate compensation for each acquisition. Samples were analysed using a BD Fortessa flow cytometer, equipped with 4 lasers (405nm, 488nm, 544nm, 633nm) and 18 detectors for fluorescent parameters. Data were analysed using FlowJo V9.9 using the gating strategy illustrated in Supplementary figure 1 and 2 and visualised using SPICE V5 ([4]) and GraphPad Prism v6.

The same gates were applied to all study visits with minor adjustments if needed. An experienced operator performed the initial gating, which was checked by another experienced operator and any queries were discussed with the scientific supervisor. Exported data were locked prior to unblinding and statistical analyses.

**Data analysis**

The following criteria were set for samples to be included in the immunogenicity analysis: a) unstimulated control was present and interpretable for each set of samples; b) frequencies of PHA- or BCG-induced total cytokine-expressing (12-hour WBA) or Ki67-expressing (7-day WBA) CD4 or CD8 T cells were greater than the median + 3MAD (median absolute deviation) of the total cytokine or Ki67-expressing CD4 or CD8 T cells of the unstimulated controls of the entire cohort; c) for each sample, the frequency of PHA- or BCG-induced total cytokine-expressing (12-hour WBA) or Ki67-expressing (7-day WBA) CD4 or CD8 T cells were greater than the frequency of the same cell population in its respective unstimulated control; d) 12-hour WBA: memory phenotype of cytokine-expressing CD4 and CD8 T cells was assessed by combined expression of CD45RA and CCR7 only if: i) more than 50 cytokine-expressing CD4 or CD8 T cells were detected upon stimulation and ii) the frequency of cytokine-expressing CD4 or CD8 T cells was 3 times greater than its respective unstimulated control; e) 7-day WBA: cytotoxic profile of Ki67-expressing (proliferating) CD4 and CD8 T cells was assessed by combined expression of perforin, granulysin, granzyme A, granzyme B and granzyme K only if: i) more than 50 Ki67-expressing CD4 or CD8 T cells were detected upon stimulation and ii) the frequency of Ki67-expressing CD4 or CD8 T cells was 3 times greater than its respective unstimulated control.

Frequencies of cytokine- and KI67-expressing cells in unstimulated control were subtracted from stimulated samples. For qualitative analyses of antigen-specific memory subsets (12-hour WBA) and cytotoxic profiles (7-day WBA) results from unstimulated control were not subtracted.

**Supplementary references**

1. Hanekom WA, Hughes J, Mavinkurve M, et al. Novel application of a whole blood intracellular cytokine detection assay to quantitate specific T-cell frequency in field studies. J. Immunol. Methods **2004**; 291:185–195.

2. Kagina BM, Mansoor N, Kpamegan EP, et al. Journal of Immunological Methods. J. Immunol. Methods **2015**; 417:22–33.

3. Soares A, Govender L, Hughes J, et al. Novel application of Ki67 to quantify antigen-specific in vitro lymphoproliferation. J. Immunol. Methods **2010**; 362:43–50.

4. Roederer M, Nozzi JL, Nason MC. SPICE: Exploration and analysis of post-cytometric complex multivariate datasets. Cytometry **2011**; 79A:167–174.

**Supplementary figure legends:**

**Supplementary figure 1: Gating strategy for 12h whole blood assay.**

A time gate was applied to ensure consistency in fluorescence during acquisition, then singlets were selected. Cells with high SSC (monocytes and granulocytes) and low FSC (debris) were excluded, CD3+ cells were selected and autofluorescent cells were excluded. CD4+ T cells were further defined as CD4+, CD8-, γδTCR- and CD56-. CD8+ T cells were further defined as CD4-, γδTCR-, CD56-, CD161low and CD8+. Gates identifying cytokine-expressing cells were set based on unstimulated (UNS) samples and kept in the same position for Ag85A and BCG stimulations. Boolean gates representing all possible combinations of IFNγ, IL2, IL17, IL22 and/or TNFα were generated to characterize T cell functional quality (as illustrated in Figure 3B). T cells expressing any of the cytokines were also identified and their memory profile was further defined as illustrated in Figure 3C.

**Supplementary figure 2: Gating strategy for 7-day whole blood assay.**

A time gate was applied to ensure consistency in fluorescence during acquisition, then singlets were selected. Dead cells were excluded, cells with low FSC (debris) and high SSC (monocytes and granulocytes) were excluded and CD3+, CD56- T cells were selected. CD4+ T cells were further defined as CD8-, γδTCR- and CD4+. CD8+ T cells were further defined as CD4-, γδTCR-, CD161low and CD8+. Gates identifying Ki67+ cells (proliferating) and those expressing cytotoxic markers were set based on unstimulated (UNS) samples and kept in the same position for Ag85A and BCG stimulations. Boolean gates representing all possible combinations of granzyme A, granzyme B, granzyme K, granulysin and perforin were generated to characterize the functional quality of Ki67+ T cells (as illustrated in Figure 4C).

**Supplementary figure 3: CD8+ T cell responses to Ag85A and BCG.**

Fresh whole blood was stimulated with Ag85A peptides **(A)** or BCG **(B)** for 12h prior to intra-cellular cytokine staining and flow cytometry. Longitudinal changes of Ag85A-specific **(A)** or BCG-specific **(B)** CD8+ T cells expressing any combination of IFNγ, TNFα, IL2, IL17 and IL22 in MVA85A (red lines and arrows) and control (black lines and arrows) arms. Medians and 95% confidence intervals (for the medians) are shown. Unadjusted p-values were calculated by mixed effects models.
